# Analysis of the Interaction between DNA Aptamers and Cytochrome C on the Surface of Lipid Films and on the MUA Monolayer: A QCM-D Study †

**DOI:** 10.3390/bios13020251

**Published:** 2023-02-09

**Authors:** Marek Tatarko, Sandro Spagnolo, Martin Csiba, Veronika Šubjaková, Tibor Hianik

**Affiliations:** Faculty of Mathematics, Physics and Informatics, Comenius University, Mlynská dolina F1, 842 48 Bratislava, Slovakia

**Keywords:** cytochrome c, lipid films, DNA aptamers, QCM-D, gold nanowires

## Abstract

We analyzed the possibility of the detection of cytochrome c (cyt c) being physically adsorbed on lipid films or covalently bounded to 11-mercapto-1-undecanoic acid (MUA) chemisorbed on the gold layer using quartz crystal microbalance with dissipation monitoring (QCM-D). The negatively charged lipid film composed of a mixture of zwitterionic DMPC and negatively charged DMPG phospholipids at a molar ratio of 1:1 allowed the formation of a stable cyt c layer. Addition of DNA aptamers specific to cyt c, however, resulted in removal of cyt c from the surface. The interaction of cyt c with the lipid film and its removal by DNA aptamers were accompanied by changes in viscoelastic properties evaluated using the Kelvin–Voigt model. Cyt c covalently bound to MUA also provided a stable protein layer already at its relatively low concentrations (0.5 μM). A decrease in the resonant frequency following the addition of gold nanowires (AuNWs) modified by DNA aptamers was observed. The interaction of aptamers with cyt c on the surface can be a combination of specific and non-specific interactions due to electrostatic forces between negatively charged DNA aptamers and positively charged cyt c.

## 1. Introduction

Cytochrome c (cyt c) is a small hemeprotein (molecular weight of 12 kDa, and size approximately 5 nm), that serves as electron carrier in a respiratory chain of the mitochondria and as an initiator of several critical processes in the living cells. Cyt c is associated with cardiolipin in the inner mitochondrial membrane and plays a key role in cellular metabolism, respiration, ATP synthesis, and protein regulation [1]. It is positively charged in physiological conditions due to the presence of lysine and arginine amino acids [2]. Cyt c is an electroactive molecule with redox properties that can be studied using electrochemical methods [3]. Cyt c is released from the mitochondrial membrane into the cytoplasm during cell apoptosis. This process is accompanied by increased permeabilization of the outer membrane of the mitochondria which is regulated by caspases [4,5]. The permeabilization of the outer membrane depends on the cell type and nature of stimuli of apoptosis, which is controlled by proteins such as Bax, Bak, and by Ca^2+^ ions [6]. Cyt c is therefore an important marker in cancer diagnostics as well as in the analysis of the effectivity of anticancer drugs in chemotherapy. Therefore, the detection of cyt c can be useful in evaluating of the effect of anticancer drugs on cancer cells [7,8]. Monitoring of the concentration of cyt c may prevent pathological conditions due to side effects. Cyt c adsorbed at the lipid membranes or organic films can also be used as a model for the study of the mechanisms in receptor–ligand interactions, as well as to study the mechanisms in the interaction between peripheral proteins and biological membranes [9,10]. Cyt c is strongly bound to negative charged lipids in membranes, mainly cardiolipin, due to electrostatic interactions [11,12]. Cyt c can be also adsorbed on carboxyl groups terminated self-assembly monolayers (SAM), for example 11-mercapto-1-undecanoic acid (MUA), via physical adsorption [13,14] or covalent binding [15]. Such a system plays an important role in molecular electronics due to the redox properties of cyt c [3].

Various standard methods, such as flow cytometry, Western blot, enzyme-linked immunosorbent assays (ELISA), and high-performance liquid chromatography (HPLC) are commonly used for cyt c detection [16]. To overcome the limitations of standard methods regarding time consumption and expensive equipment, the development of biosensors opens novel approaches to faster and easier evaluation. Several biosensors based on DNA aptamers as recognition elements have been reported so far using various methods of cyt c detection [12,17,18,19,20,21,22]. Detection of cyt c using biosensors based on antibodies has also been reported [16]. However, substantial attention in recent years has been focused on the application of nucleic acid aptamers (DNA or RNA) in biosensor development. Most works use DNA aptamers due to their better stability in comparison with RNA.

DNA aptamers are single stranded molecules that can bind a target with high specificity. They can be developed in vitro against practically unlimited types of targets, such as toxins, antibiotics, proteins, whole cells, bacteria, viruses, etc., using combinatorial chemistry known as SELEX (systematic evolution of ligands by exponential enrichment). DNA aptamers have great potential in biosensor development [23] as well as in targeted therapy [24] due to their easy chemical modification, stability, and a negligible immunogenicity. In a solution, they fold into a 3D structure forming a binding site for the target [25].

However, there is limited research on the interaction between DNA aptamers and cyt c on the lipid membranes and organic layers. In particular, we applied single molecule force spectroscopy (SMFS) using DNA aptamers covalently immobilized at the AFM tip to study its interaction with cyt c immobilized at DMPC bilayers modified by calixarenes at the mica surface [26].

Nanostructures, such as gold nanoparticles (AuNPs) or gold nanowires (AuNWs) modified by DNA aptamers, can be effectively used as a drug carrier for targeted delivery as well as for signal amplification in biosensors. Nanowires can be also propelled by chemical or external sources, such as ultrasound, and thus serve as nanomotors. Functionalization of their surface by antibodies or nucleic acid aptamers allows them to accelerate the delivery of therapeutic agents and diagnostics [27]. In this respect, the system of cyt c adsorbed at supported lipid membranes can serve as a model for the study of the interaction between aptamer-modified nanostructures and cell membrane receptors. This is important for the development of biosensors that can detect cancer cells, such as leukemic or breast cancer cells. For example, the aptamers that selectively bind to the protein tyrosine kinase 7 (PTK7)—the cancer marker that is localized in the membrane of various cancer cells—were used to detect leukemic cells using electrochemical [28] and acoustic methods [29]. For amplification of detection, the nanoparticles modified by aptamers can be rather useful. This has been demonstrated in our work focused on the development of acoustic biosensors for the detection breast cancer cells [30]. However, further research is necessary to optimize this amplified detection.

Despite the good sensitivity of electrochemical methods of cyt c detection, we demonstrated that the QCM-D method has the advantage of direct monitoring of sensor preparation, including the adsorption of cyt c onto the lipid films and MUA layers. Moreover, the interaction of AuNWs modified by aptamers has been directly monitored in real time. In addition, the multiharmonic QCM-D method allowed us to not only to analyze the frequency and dissipation changes, but also to determine important viscoelastic properties of the system, such as penetration depth of acoustic wave, *Γ*, viscosity coefficient, *η*, and shear modulus of elasticity, *μ*. These values are important for analysis of the binding properties of the target to the sensing surface.

In this work, the multiharmonic quartz crystal microbalance with dissipation monitoring (QCM-D) has been applied to study the interaction between cyt c and supported lipid films and DNA aptamers specific to this protein. Cyt c has also been covalently immobilized at the surface of the monolayer of MUA, and the interaction between DNA aptamers and those immobilized on AuNWs were analyzed. In addition, using the Kelvin–Voigt model [31], we evaluated the viscoelastic properties of supported lipid films with various modifications.

## 2. Materials and Methods

### 2.1. Chemicals

For liposome and MUA monolayer preparation, the following chemicals were used: PBS (phosphate buffered saline composed of 10 mM Na_2_HPO_4_, 1.8 mM KH_2_ PO_4_, 137 mM NaCl, 2.7 mM KCl and 2 mM CaCl_2_ diluted in MiliQ water, pH 7.4) and MiliQ water with a resistance of 18 MΩ.cm was prepared by Purelab Classic UV (Elga, High Wycombe, UK). The standard chemicals such as ethanol, NaCl, HNO_3_, NH_3_, H_2_O_2_, MgCl_2_, and CaCl_2_ were purchased from Slavus (Bratislava, Slovakia). Liposome solution was prepared from 1,2-dimyristoyl-sn-glycero-3-phosphocholine—DMPC (Avanti Polar Lipids Inc., Birmingham, AL, USA) and 1,2-Dimyristoyl-sn-glycero-3-phospho-rac-(1-glycerol) sodium salt—DMPG (Sigma Aldrich, Darmstadt, Germany). 1-dodecanethiol (DDT) (Sigma Aldrich, Darmstadt, Germany) was used to modify the gold layer on the quartz crystal surface. For covalent binding of cyt c, 11-mercapto-1-undecanoic acid (MUA) and bovine serum albumin (BSA) (Sigma Aldrich, Darmstadt, Germany) were applied.

Gold plating solution OROTEMP and silver plating solution TECHNI SILVER CY LESS II W (Italgalvano, Lodi, Italy), alumina powder (Schmitz-Metallographie GmbH, Herzogenrath, Germany), methylene chloride (Sigma-Aldrich, Darmstadt, Germany), nitric acid, isopropanol, ethanol (Slavus, Bratislava, Slovakia). N-(3-Dimethylaminopropyl)-N′-ethylcarbodiimide hydrochloride (EDC), N-Hydroxysuccinimide (NHS), and MES hydrate were purchased from Sigma Aldrich, Darmstadt, Germany.

DNA aptamer sensitive to cyt c was purchased from Biosearch Technologies (Risskov, Denmark). The aptamer (NH_2_-Apt-cytc) of the following sequence: 5′-NH_2_-TTTTTTTTTTATCGATAAGCTTCCAGAGCCGTGTCTGGGGCCGACCGGCGCATTGGGTACGTTGTTGCCGTAGAATTCCTGCAGCC-3′ was modified at the 5′ end by amino group [32]. A 10-mer thymidine spacer at the 5′ end was used for providing better aptamer flexibility. We also used a DNA aptamer that does not specifically bind to cyt c with the following sequence: 5′-NH_2_-CTGAATTGGATCTCTCTTCTTGAGCGATCTCCACA-3′. This DNA aptamer was purchased from Generi Biotech Ltd. (Hradec Králové, Czech Republic).

### 2.2. Synthesis of Gold Nanowires

The gold nanowires (AuNWs) were synthesized using a common template assisted by electrochemical deposition into a polycarbonate (PC) membrane with pore size 400 nm (Whatman, Inc., Dassel, Germany). We used a 3-electrode system in a home-made Teflon cell, volume 5 mL [27]. The PC membrane with a thin gold layer (50 nm) served as a working electrode, Ag/AgCl was used as a reference electrode, and a platinum electrode as an auxiliary electrode (CH Instruments, Inc., Austin, TX, USA). The electrodes were placed into the Teflon cell with silver plating solution to form a sacrificial layer at a potential of −0.9 V vs. Ag/AgCl and at charge of 0.1 C. After washing the membrane with MilliQ water, the Au was deposited using the gold plating solution with potential of −1 V and at the charge of 1.5 C. Then the membrane was rinsed with MilliQ water. The free AuNWs were subsequently released from the PC membrane using mechanical and chemical cleaning by polishing with alumina powder (3–4 μm particles size) and then the membrane was exposed to nitric acid (8 M HNO_3_), respectively. Subsequently, the PC membrane was dissolved in methylene chloride and mixed thoroughly for 30 min in a vortex and 15 min in an ultrasonic bath. AuNWs were then separated from the solution by centrifugation at 8000 rpm for 5 min and washed repeatedly with isopropanol, ethanol, and MilliQ water at 8000 rpm for 3 min. The AuNWs were stored in MilliQ water in the refrigerator [33]. The dimension of AuNWs was determined by electron microscope. The length of AuNWs was determined as 3.22 ± 0.38 μm. The diameter of the AuNWs of approximately 400 nm corresponded to the pore size of the PC membrane. The electron microscopy image of AuNWs is presented in Figure S1 of Supplementary Materials.

### 2.3. Functionalization of AuNWs with DNA Aptamers

**Figure 1:**
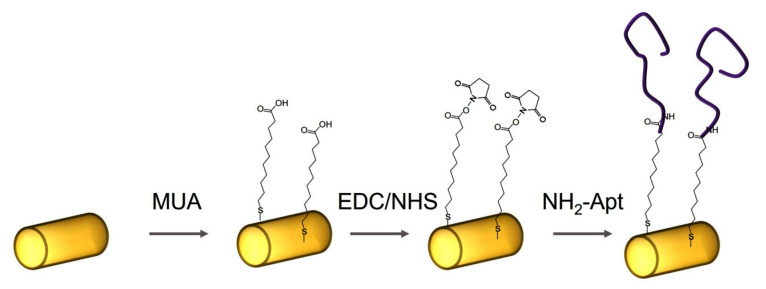
The scheme of functionalization of AuNWs using amino-modified DNA aptamers specific to cyt c (NH2-Apt).

Functionalization of AuNWs with DNA aptamers was performed via carbodiimide coupling [34]. First, bare AuNWs were immersed in 20 mM solution of 11-mercapto-1-decanoic acid (MUA) in ethanol for 30 min, thus forming a self-assembled layer on the gold surface through the thiol group. Then, MUA-modified AuNWs were centrifugated twice (8000 rpm, 3 min) and washed by MilliQ water. To activate the carboxyl group, MUA-modified AuNWs were then incubated for 1 h in an EDC/NHS solution (400 mM EDC and 100 mM NHS in an MES buffer solution, pH 6.5). MES is preferred for coupling EDC/NHS because it is a non-amine and non-carboxylate buffer that does not interfere with EDC due to competition for activation [35]. Finally, after washing with MilliQ water to remove unbound components, the activated surface of AuNWs were immersed in DNA aptamer solution (1 μM NH_2_-Apt-cytc in 10 mM PBS) for 1 h. Finally, DNA aptamer functionalized AuNWs were centrifugated twice (8000 rpm, 3 min) and washed in working buffer (10 mM PBS containing 2 mM CaCl_2,_ pH 7.4). All steps of functionalization of AuNWs surfaces were performed at room temperature. The scheme of functionalization of AuNWs using DNA aptamers is presented in Figure 1.

### 2.4. Piezocrystal Cleaning, Preparation of Lipid Films, and MUA Monolayers with Covalently Attached Cyt C

Quartz crystals (Total Frequency Control, Storrington, UK), fundamental frequency 8 MHz, working area 0.2 cm^2^ consisted of a thin gold layer, were cleaned before each measurement. Crystals were submerged in basic Piranha solution (29% NH_3_, 30% H_2_O and H_2_O_2_ with volumetric ratio 1:5:1, respectively) for 25 min. After this treatment, the crystal was washed 3 times with deionized water and stored in ethanol. The crystal was then submerged in 2 mM solution of 1-dodecanthiol (DDT) in ethanol and stored at room temperature for 16 h. After washing with ethanol and drying in a flow of nitrogen, the crystal was placed in an acryl flow cell (JKU Linz, Austria) connected to the syringe pump (Genie Plus, Kent Scientific, Torrington, CT, USA). 

**Figure 2:**
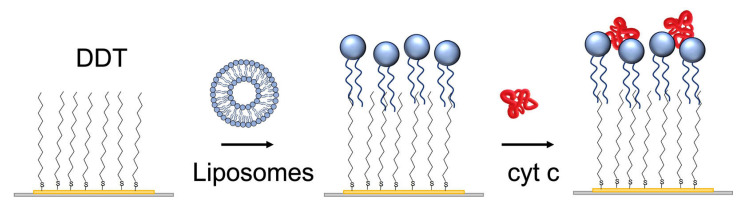
The scheme of preparation of the lipid film at the surface of the 1-dodecanethiol (DDT) layer chemisorbed at the gold surface of the piezocrystal by liposome fusion. Finally, cyt c was adsorbed.

The lipid film was prepared using liposome fusion. For this purpose, the small liposomes were prepared using the sonication method. Briefly, 8 mg of the phospholipid mixture (DMPC: DMPG in a molar ratio of 1:1) was dissolved in a small quantity of chloroform and dried under nitrogen in order to deposit a layer on the walls of the flask. A 4 mL aliquot of PBS was then added, and after a 30 min incubation, the mixture was ultrasonicated for 20 min with a sonicator (Bandelin Sonorex RK31, Berlin, Germany) in a water bath at room temperature [36]. The liposomes in a concentration of 0.5 mg/mL were then added in a flow to a gold layer of quartz crystal modified by DDT (Figure 2).

**Figure 3:**
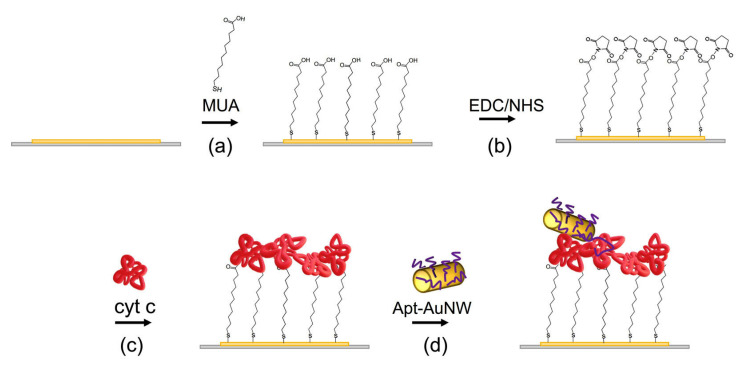
The scheme of preparation of the MUA layer with covalently immobilized cyt c and the interaction with AuNWs modified by DNA aptamers. (**a**) MUA is chemisorbed at the thin gold layer of the piezocrystal; (**b**) terminal carboxyl groups of the MUA are activated by EDC/NHS; (**c**) cyt c is added and binds to the activated carboxyl groups of the MUA; (**d**) AuNWs modified by DNA aptamers specific to cyt c are added and interact with cyt c.

The MUA self-assembled monolayers were prepared by chemisorption at the clean gold layers. Crystals were incubated in 2 mM solution of MUA dissolved in ethanol overnight. Crystals were then rinsed with MiliQ water and incubated for 35 min in a mixture of 20 mM EDC and 50 mM NHS. The crystals were then rinsed, dried under the nitrogen stream, and placed into the flow cell. The scheme of the MUA layer with covalently immobilized cyt c and the addition of AuNWs modified by DNA aptamers is presented in Figure 3.

### 2.5. The Principles of QCM-D and Viscoelastic Properties of Lipid Layers with Cyt C

The principle of the QCM-D consists of measurements of the changes in the resonant frequency that are related to the changes of the mass on the crystal surface. According to Sauerbrey [37], the changes in the resonant frequency, Δ*f*, of the quartz crystal in the vacuum are related to the changes in mass, Δ*m*, by equation:

(1) $$\Delta f=\frac{-2nf_{0}^{2}\Delta m}{A\sqrt{\mu_{q}\rho_{q}}}$$

where *n* is the harmonic number, *f*_0_ is the fundamental resonance frequency, *A* is the effective crystal area, *μ_q_ =* 2.947 × 10^11^ g·cm^−1^·s^−2^ is the shear modulus of elasticity, and *ρ_q_* = 2.648 g·cm^−3^ is the crystal density. In a water environment, the frequency can be affected by viscous forces, therefore additional terms should be added to the Sauerbrey equation:

(2) $$\Delta f=2f_{0}^{\frac{3}{2}}\sqrt{\frac{\eta_{L}\rho_{L}}{\pi {µ}_{q}\rho_{q}}}$$

where *η_L_* is the viscosity and *ρ_L_* is the density of the liquid, respectively [28]. The acoustic waves in QCM transducer are generated by applying a high-frequency voltage to the electrodes sputtered at both sides of the crystal [38,39,40]. The attenuation of acoustic waves due to viscous forces is characterized by changes in penetration depth (decay length), *Γ,* and dissipation, *D*. Decay length is related to the viscosity, *η_L_*, and density, *ρ_L_*, of the aqueous solution as follows:

(3) $$\Gamma =\sqrt{\frac{2\eta_{L}}{\omega \rho_{L}}}$$

where ω is the circular frequency. Dissipation can be described as:

(4) $$D=\frac{2\Gamma }{f_{0}}$$

The QCM-D experiments were created by the computer-controlled Sark 110 vector analyzer (Seeed, Shenzhen, China). This device allowed measurement of fundamental and higher harmonic frequencies. The frequency changes increased linearly with the harmonic number, *n* (see Equation (1)). All measurements were conducted in flow mode at room temperature at approximately 20 ± 0.5 °C.

Using the frequency and dissipation data, it is possible to estimate the viscoelastic values of the layers at the surface of the piezocrystal. It is, however, useful to verify whether this evaluation correctly reflects the viscoelastic properties. For this purpose, the analysis of the normalized frequencies for several overtones *f_n_/n* and their relative changes Δ*f_n_/n* are helpful. The more these values differ from each other, the higher the viscoelastic component of the sample is. By means of viscoelastic analysis, it is possible to obtain information about the characteristics of the sensing layer, such as viscosity and elasticity. Using the Kelvin–Voigt viscoelastic model, it is possible to analyze the viscoelastic properties of the adlayer considering the differences in frequency and dissipation at the different harmonics. According to Voinova et al. [31], the viscoelastic properties of the films are related to the variations in frequency and dissipation:

(5) $$\Delta f\approx -\frac{h_{1}\rho_{1}\omega }{2\pi \rho_{0}h_{0}}\;\left(1+\frac{h_{1}^{2}\chi }{3\Gamma^{2}\left(1+\chi^{2}\right)}\right)$$

(6) $$\Delta D\approx \frac{2h_{1}^{3}\rho_{1}\omega }{3\pi f_{0}\rho_{0}h_{0}}\frac{1}{\Gamma^{2}\left(1+\chi^{2}\right)}$$

where 𝜒 = $\frac{\mu_{1}}{\eta_{1}\omega },$ *Γ* is the decay length of the shear wave in the liquid medium, $\rho_{0}$ is the density of quartz (2.648 g·cm^−3^), $h_{0}$ is the thickness of the quartz crystal (0.208 mm), $h_{1}$, $\mu_{1}$, $\eta_{1}$, and $\rho_{1}$ are the thickness, the shear elastic modulus, the viscosity, and the density of the adsorbed film, and *ω* is the angular frequency of oscillation (equivalent to 2π*f*). 

## 3. Results and Discussion

### 3.1. The Interaction of Cytochrome C with Supported Lipid Films and with DNA Aptamers

**Figure 4:**
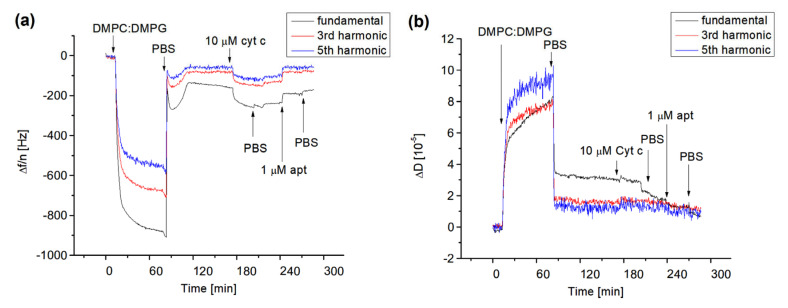
The kinetics of the changes in the resonant frequency divided by the number of harmonics, Δ*f/n*, (**a**) and dissipation, Δ*D*, (**b**) for fundamental frequency, third and fifth harmonics following the addition of DMPC:DMPG (1:1) liposomes, 10 μM cyt c and 1 μM aptamer (apt) specific to cyt c on 1-dodecanthiol layer chemisorbed on the thin gold layer of piezocrystal. The moments of addition of liposomes, cyt c, DNA aptamer, and PBS wash are shown by arrows.

In the first series of experiments, we studied the formation of lipid films on the surface of 1-dodecanethiol (DDT) self-assembled monolayers (SAM) chemisorbed on the thin gold layer of quartz crystal with a fundamental resonant frequency of 8 MHz. The lipid films were formed by the fusion of small liposomes composed of various molar ratios of DMPC and DMPG. DMPC and DMPG are frequently used for preparation of lipid films mimicking the lipid bilayer of biomembranes. As DMPG promotes a negative charge, it accelerates the electrostatic interaction of cyt c with the lipids. The Sark 110 vector analyzer was used to monitor the changes in the fundamental resonant frequency, Δ*f*, and their harmonics, as well as for evaluation of the dissipation changes, Δ*D*. First, it was necessary to optimize the buffer composition for preparation of the lipid films and the optimal molar ratio of DMPC:DMPG for adsorption of cyt c. We found that at the molar ratio of DMPC:DMPG = 1:1, the positively charged cyt c was physically adsorbed into the surface of the lipid film due to the negatively charged DMPG. However, for pure DMPC and DMPC:DMPG = 10:1, no significant changes in the resonant frequency occurred following the addition of cyt c up to 10 μM (results not shown). We also found that for the formation of the supported lipid films, the PBS containing 2 mM CaCl_2_ is advantageous. The positive effect of calcium for formation of the supported lipid film has been demonstrated earlier by Mirsky et al. [36]. Figure 4 shows the typical kinetics of the changes in fundamental resonant frequency (Δ*f*), dissipation (Δ*D*), and normalized third (24 MHz) and fifth (40 MHz) harmonics following the formation of the lipid film, adsorption of cyt c, and the addition of DNA aptamers specific to cyt c.

As can be seen from Figure 4a, the addition of liposomes on the surface of DDT with a flow rate 50 μL/min resulted in a sharp decrease in the resonant frequency and their harmonics. Significant differences between the normalized fundamental frequency and their harmonics are also evident. This is due to the viscosity contribution as demonstrated in Figure 4b, where the kinetics of dissipation changes are presented. The washing of the surface by PBS resulted in an increase in resonant frequency and decrease in dissipation. Furthermore, the values of normalized fundamental frequencies and their harmonics become closer to each other. This means that the viscoelastic contribution is less expressed. We assume that the interaction between liposomes and the DDT layer resulted in the formation of lipid multilayers. The washing of the surface by PBS caused the removal of less adjacent lipid films. The overall change in fundamental frequency following the formation of the lipid film after the PBS wash was approximately −150 Hz. The maintenance of an almost steady-state level of the fundamental frequency, their harmonics, and dissipation is evidence of formation of the stable lipid film. Using the frequency changes and Sauerbrey Equation (1), one can estimate the surface density of the phospholipids as follows: *σ* = ∆*mN_A_*/(*A* × *Mw*), where *N_A_* = 6.022 × 10^23^ mol^−1^ is Avogadro’s number and *Mw* = 672.42 g/mol is average molecular weight of DMPC and DMPG. The estimation gives for the lipid film the value Δ*m*/*A* = 9.27 × 10^14^ molecules/cm^2^. The average area per DMPC and DMPG molecules estimated from Langmuir isotherms of lipid monolayers in a condensed state at pH 7 is 0.59 nm^2^ = 5.9 × 10^−13^ cm^2^ [41]. Then the surface density of phospholipids is 1.69 × 10^14^ molecules/cm^2^. This value is approximately 5.48 times lower in comparison with those estimated from QCM-D data. Therefore, assuming that the contribution of the viscosity to the frequency changes is not dominant as is evident from changes of dissipation, one can conclude that instead of the lipid monolayer, the lipid multilayers are adsorbed on the DDT surface. The number of monolayers can be approximately five as it follows from the ratio of the surface density obtained from QCM-D experiments and lipid monolayers. In this case, the first monolayer closer to the DDT surface is stabilized by hydrophobic interactions between hydrocarbon chains of phospholipids and alkyl chains of DDT. The other four monolayers probably form two lipid bilayers stabilized by calcium ions presented in PBS.

The addition of 10 μM cyt c at a flow rate of 50 μL/min also decreased the resonant frequency and their harmonics but only slightly affected the dissipation, especially for higher harmonics (Figure 4). This is evidence of physical adsorption of positively charged cyt c to negatively charged lipid film. Cyt c probably forms a relatively rigid protein layer. The final change in fundamental frequency after washing of the surface by PBS caused by cyt c is −77 Hz. Using a similar approach as above and considering that the molecular weight of cyt c is 12,000 g/mol (12 kDa), one can obtain the surface density of cyt c: 2.67 × 10^13^ molecules/cm^2^. As it follows from the crystallographic data, the hydrodynamic radius of free cyt c is r_H_ = 1.7 nm [42]. This corresponds to the cyt c area of approximately 9.07 nm^2^. Thus, the maximal surface density of cyt c at the protein film can be estimated as 1.1 × 10^13^ molecules/cm^2^. This value is approximately 2.4 times lower than those obtained from QCM-D experiments. It is likely that cyt c does not form the monolayer, but at least two protein layers. In addition, one can consider that adsorption of cyt c at the surface may change its conformation which can result in an increase in the molecule dimensions. This has been demonstrated in a paper by Ghosh et al. [42], which studied the adsorption of cyt c on the gold nanoparticles and showed an increase in r_H_ from 1.7 to 2.4 nm, i.e., 1.4 times. The addition of the DNA aptamer in flow mode at the same rate as those for cyt c resulted in an increase in resonant frequency and their harmonics as well as a decrease in dissipation. This is clear evidence of removal of cyt c from the surface of the lipid film. The DNA aptamer specific to the cyt c should form complexes with the cyt c. Most probably there is competence between the binding of cyt c to the DNA aptamer with those of the phospholipids. The increase in the frequency is evidence that the complexes between cyt c and DNA aptamers are preferable. The final frequency changes following the addition of the 1 μM DNA aptamer are 64 Hz. This value is close to those resulting from the adsorption of cyt c into the lipid surface. This means that DNA aptamers, even at lower concentration than cyt c, removed practically all cyt c from the surface of the lipid film. It is possible that the binding stoichiometry between cyt c and the DNA aptamer is higher than one. We can speculate that due to the negatively charged DNA aptamer, there is also a non-specific interaction between the positively charged cyt c and DNA. To further analyze this effect, we also used a non-specific DNA aptamer in the same concentration (1 μM). The addition of the non-specific DNA aptamer to the surface of the lipid film with adsorbed cyt c (10 μM) resulted in a comparable increase in the frequency, as was observed for the specific DNA aptamer (results not shown).

To obtain additional insight on the mechanisms of interaction of cyt c and DNA aptamers with the lipid films, we analyzed changes in penetration depth, Δ*Γ*, viscosity coefficient, Δ*η*, and shear modulus of elasticity, Δ*μ*. These values were obtained using the data presented in Figure 4 and the Equations (5) and (6) are presented in Table 1.

The penetration depth, *Γ*, can be described as the distance travelled by the acoustic wave from the surface of the crystal in the aqueous medium. According to Equation (3) it is affected by the viscosity, *η_L_*, and density, *ρ_L_*, of the liquid surrounding the adlayer at the surface of the piezocrystal. Changes in the penetration depth can be affected by the properties of the adsorbed lipid film and the aqueous medium. According to our estimations, the addition of liposomes on the surface of the DDT layer resulted in substantial decay of penetration depth by 148 nm. This is clear evidence of the increase in dissipation (and hence the viscosity contribution), as can be seen in Figure 4b. 

The propagation of the acoustic wave is suppressed due to the viscosity effect. However, after washing of the surface by PBS, the changes in penetration depth are lower at 19.4 nm. This can be explained by the formation of a relatively rigid film due to the removal of softer multilayers from the crystal surface. Please note that the penetration depth represents only the properties of the lipid film, but not the film thickness. A similar effect was observed following the adsorption of cyt c. After washing of the surface by PBS, the changes in *Γ* were calculated as 11.7 nm. Thus, cyt c affects the penetration depth only slightly (by 7.7 nm) compared to the lipid film. A substantial increase in the changes in penetration depth were observed following the addition of DNA aptamers: Δ*Γ* = 61.6 nm. This can be explained by the interaction between the aptamer and cyt c, but also with the lipid film. The depression of the propagation of the penetration depth in the latter case may be due to an increase in the viscosity coefficient (Table 1) by 1.53 mPa·s. This is much higher in comparison with the increase in the viscosity coefficient following the formation of the lipid film (0.1 mPa·s) and cyt c layer (0.05 mPa·s). The observed changes are substantially lower in comparison with those of the formation film of histones reported by Dutta et al. [45] which caused an increase in the viscosity coefficient by 1.2 mPa·s. Comparable changes in the viscosity coefficient have been reported in our recent work by Spagnolo et al. [46] for β-casein layers at the DDT: Δ*η* = 0.96 mPa·s. However, cyt c is a much smaller protein compared to histones and β-casein and forms more rigid layers. 

We also estimated the changes in the shear elasticity modulus, Δ*μ*. The formation of the lipid layer causes an increase in this value up to 5.72 × 10^5^ Pa. The formation of cyt c layer caused an increase in the elasticity modulus of up to 5.63 × 10^5^ Pa. This is clear evidence of the absorption of the protein to the lipid layer which affects its mechanical properties. The increase in elasticity modulus is not only due to the formation of a relatively rigid protein layer but also due to an increase in the ordering of the polar part of the phospholipids due to electrostatic attractive forces. The increase in the elasticity modulus, Δ*μ* = 4.91 × 10^5^ Pa, has been observed after the interaction of DNA aptamers with the lipid film modified by cyt c. This suggests that aptamers interact with the cyt c and probably also with the lipid film. The observed increase in elasticity modulus, increase in viscosity coefficient, and changes in penetration depth are evidence of the rather complex behavior of the system studied. The possible partial removal of cyt c from the surface of the lipid film following interaction with DNA aptamers as well as the comparable effect caused by non-specific DNA aptamers make this system rather complicated for study of the specific interaction between aptamers and cyt c on the surface. The positively charged cyt c is adsorbed on the lipid film due to the electrostatic interaction with the negatively charged DMPG. These interactions, although stronger than van der Walls forces, cannot provide a stable system following the addition of negatively charged DNA aptamers, which compete with the electrostatic forces that stabilized the cyt c film on the lipid surface. We therefore analyzed the interaction between DNA aptamers and cyt c on the surface of 11-mercapto-1-undecanoic layer (MUA), onto which the protein has been covalently attached. For amplification of cyt c detection using the QCM-D method, we also used gold nanowires (AuNWs) onto which the amino-modified DNA aptamers specific to cyt c were covalently attached using NHS/EDC chemistry.

### 3.2. The Interaction of DNA Aptamers with Cytochrome C Covalently Attached to the 11-Mercaptoundecanoic Layer

**Figure 5:**
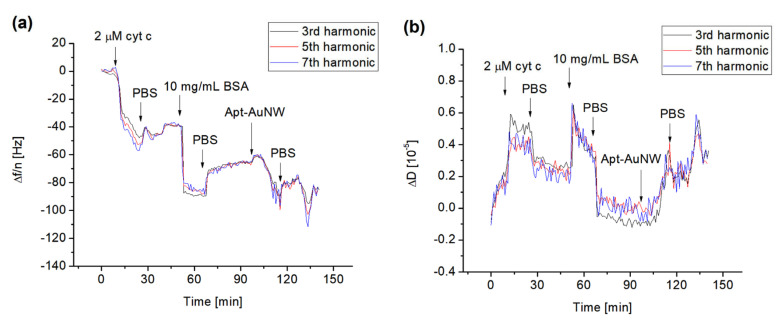
The kinetics of the changes in normalized resonant frequency (**a**) and dissipation (**b**) for third (24 MHz), fifth (40 MHz) and seventh (56 MHz) harmonic frequencies vs. time following addition to the MUA surface of 2 μM cyt c, 10 mg/mL BSA, and AuNW modified with DNA aptamers specific to cyt c (Apt-AuNW). The moments of addition of cyt c, BSA, Apt-AuNW, and PBS wash are shown by arrows.

In analogy with previous experiments, we used the QCM-D method to study the formation of the cyt c layer by its covalent immobilization at the surface of the MUA. We also analyzed the interaction of free DNA aptamers and those covalently attached to the AuNWs. The typical changes in the normalized higher harmonics, Δ*f/n*, and dissipation, *ΔD*, following the addition of cyt c to the surface of the MUA with activated carboxyl groups using EDC/NHS are presented in Figure 5. The addition of 2 μM of cyt c resulted in a decrease in the resonant frequency that after washing with PBS is stabilized. For example, the changes in the normalized third harmonics were in this case −40 Hz. It can also be seen that the frequency changes for the harmonics are close to each other. This is evidence of a weak effect of viscosity and formation of a relatively rigid cyt c layer. This also means that the Sauerbrey Equation (1) can be used for calculation of the mass density. To block the possible free binding site of MUA, we used BSA in a concentration of 10 mg/mL. As can be seen from Figure 5, the addition of BSA and subsequent washing of the surface by PBS resulted in a further decrease in the frequency and increase in dissipation. The addition of AuNWs modified by DNA aptamers resulted in a further decrease in the frequency. This is evidence of the interaction between DNA aptamers and cyt c. In independent experiments, we also applied AuNWs without aptamers. In this case, no changes in frequency and dissipation occurred (See Figure S2 of Supplementary Materials). Addition of 1 μM DNA aptamer to the cyt c immobilized on the MUA layer also resulted in a frequency decrease, but the changes are lower in comparison with those caused by aptamers immobilized at the AuNWs (see Figure S3 of Supplementary Materials). Thus, AuNWs modified by DNA aptamers can be used as effective tools for amplification of detection of cyt c on surfaces.

**Figure 6:**
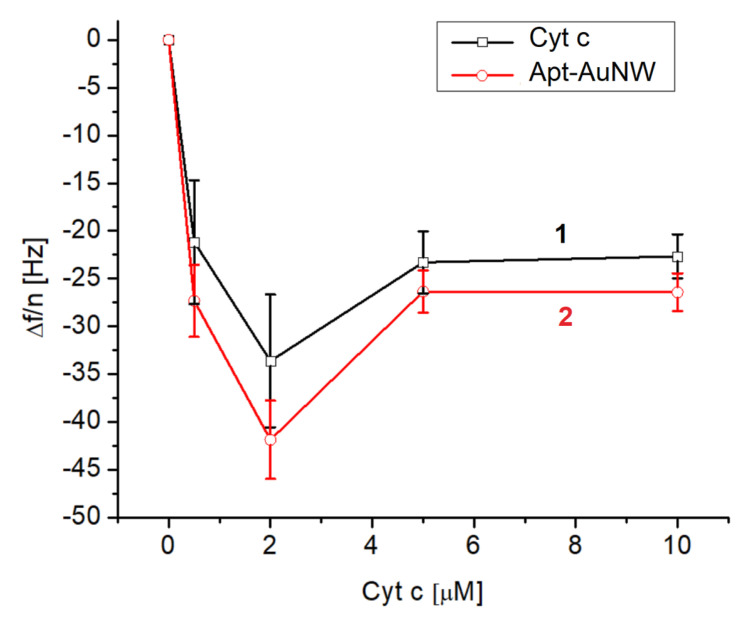
The plot of the normalized third harmonic frequency vs. concentration of cyt c on the surface of MUA activated by EDC/NHS (curve 1) and following the addition of AuNWs modified by DNA aptamers (concentration of DNA aptamer was 1 μM before centrifugation and washing of AuNWs) (curve 2). Results are mean ± SD obtained from three independent experiments.

The same approach was used for measurements of the changes in frequency and dissipation following the addition of various concentrations of cyt c in a range of 0.5–10 μM. The changes in the normalized third harmonic vs. the concentration of cyt c without and with AuNWs modified by DNA aptamers are presented in Figure 6. It is seen that an already relatively low concentration of cyt c (0.5 μM) causes rather significant frequency changes: –21.16 ± 6.48 Hz. It can also be seen that the highest frequency changes were observed for 2 μM cyt c. We assume that a lower concentration of cyt c is not sufficient to fully cover the whole crystal surface. Certainly, using the frequency changes and Sauerbrey Equation (1), one can estimate the thickness and surface coverage of cyt c at its various concentrations. These values are presented in Table 2.

Lower changes in the frequency at higher concentrations of cyt c (>2 μM) can be explained by saturation of the surface by cyt c already at its concentration of 2 μM. Further increases in cyt c concentration therefore do not contribute to the frequency changes. It is interesting to compare the surface density of cyt c on the MUA and on the lipid film. As can be seen in Table 2 and from the calculation of surface density for a 10 μM concentration of cyt c (See Section 3.1), the surface density of cyt c on MUA layers is approximately 3.4 times lower. This surface density is also slightly lower (by 1.4 times) in comparison with the maximal possible surface density of cyt c considering the crystallographic data. This means that cyt c at the surface of MUA certainly forms a protein monolayer. As it is also evident from Figure 6 (curve 2) higher adsorption of cyt c corelates with the higher frequency response following the addition of AuNWs modified by DNA aptamers. At the same time, rather low values of thickness also suggest formation of the cyt c monolayer on the MUA. The thickness is, however, lower than the diameter of cyt c. This suggests possible conformational changes in this protein on the MUA surface.

The formation of cyt c-poly (aniline sulfonic acid) (PASA) multilayers on an MUA surface has been studied in detail by Kepplinger et al. [47] using the electrochemical QCM-D method. They showed that cyt c—PASA forms rigid layers. They also determined the surface density of the cyt c monolayer at MUA. For 23 °C this corresponds to 4.8 × 10^12^ molecules/cm^2^, which is close to the values obtained in our work.

## 4. Conclusions

In this work we applied a quartz crystal microbalance with dissipation monitoring (QCM-D) for analysis of the interaction of DNA aptamers with cytochrome c (cyt c) physically adsorbed on supported lipid films. We have shown that the liposome fusion onto the 1-dodecanethiol (DDT) monolayer chemisorbed at the gold surface of a quartz crystal provides stable lipid films. However, instead of a lipid monolayer, the multilayers are formed. At the optimal molar ratio (1:1) of zwitterionic (DMPC) and negatively charged (DMPG) phospholipids, the cyt c forms a relatively rigid monolayer at the surface of the lipid film. However, the addition of DNA aptamers resulted in the removal of cyt c from the surface. This can be due to the formation of the complexes of cyt c with DNA aptamers. This suggests that the interaction between DNA aptamers and cyt c is combination of specific binding and non-specific electrostatic interactions between DNA aptamers and cyt c. This has been approved by using a non-specific DNA aptamer, which also the caused removal of cyt c from the surface. These processes were characterized by significant changes in viscoelastic properties analyzed by the Kelvin–Voigt model. We have shown that the lipid layer is well packed on the surface of the QCM disc. The cyt c binds quite strongly to the negatively charged lipid layer and forms a rather rigid protein film. Thus, the supported lipid films allow us to obtain an important insight into the interaction between cyt c and the lipids. However, the mechanisms of interaction between DNA aptamers and cyt c on the lipid film surface is complicated. It is likely that the DNA aptamer originally developed for binding cyt c in a volume has a different affinity to the protein adsorbed at the surface.

To provide a more stable cyt c layer, we also immobilized this protein covalently onto the surface of the monolayer formed by 11-mercapto-1-undecanoic acid (MUA) chemisorbed at the gold surface of the piezocrystal. In this case, the addition of either free aptamers or those immobilized at gold nanowires (AuNWs) resulted in a decrease in the resonant frequency, confirming binding to cyt c. The AuNWs modified by DNA aptamers allowed us to amplify the frequency changes. We have shown that cyt c at relatively small concentrations (0.5 μM) induced rather large changes in resonant frequency (−21.16 ± 6.48 Hz). With increased concentration of cyt c, the saturation of the surface at 2 μM cyt c took place. Using QCM-D data and the Sauerbrey equation, we estimated that the surface density of cyt c that was lower in comparison with the maximally available surface density in the case of a fully occupied surface.

The obtained results provide new insight into the interaction between DNA aptamers and cyt c on the surface of lipid films. The important conclusion from this research is the fact that the detection of the physically adsorbed proteins at the lipid films by the DNA aptamers is accompanied by the removal of aptamer–protein complexes. At the same time, further effort is necessary for selection of the aptamers to the proteins adsorbed at the surfaces, as the affinity of the aptamers originally selected for free proteins in a volume can be different. The results of the detection of the cyt c covalently attached to the MUA layers using AuNWs modified by aptamers show that these nanostructures can be used for amplification of detection of the membrane proteins, such as cancer markers for early diagnostics of cancer diseases. 

The performed research can be considered as a first step in the understanding of the mechanisms of the interaction between DNA aptamers and cyt c on these surfaces. More effort is required to obtain a better understanding of these processes. Further research could be focused on the involvement of cardiolipin in the formation of lipid films for better modeling of the mitochondrial membrane. It would be also challenging to study the mechanisms of interaction of aptamers with supported lipid films containing integral proteins. These proteins, in contrast with cyt c, more strongly interact with the lipid membrane.

## Figures and Tables

**Table 1:** The changes in normalized frequency corresponding to the fundamental resonant frequency, Δ*f*, changes in dissipation, Δ*D*, changes in mass density, Δ*m/A,* determined from Sauerbrey equation, thickness, *h*, and surface density, *σ*, of the adlayers on a DDT surface. The thickness has been calculated based on the density of the DMPC bilayers, 1.02 g/cm^3^ [43] and cyc c, 1.35 g/cm^3^ [44]. The changes in penetration depth, Δ*Γ*, viscosity coefficient, Δ*η*, and shear modulus of elasticity, Δ*μ*, of the adlayers after washing with PBS calculated using Equations (5) and (6) based on the data presented in Figure 4.

Parameter	Composition of the Adlayer		
	Lipid Film	Cyt C	DNA Aptamer
Δ*f,* Hz	−150	−77	70
Δ*D,* 10^−5^	3.17	1.18	1.11
Δ*m/A,* ng/cm^2^	1035	531.3	−483
*h,* nm	10.14	3.9	3.3
*σ,* 10^13^ cm^−2^	92.7	2.67	-
Δ*Γ*, nm	19.4	11.7	61.6
Δ*η*, mPa·s	0.1	0.05	1.53
Δ*μ*, 10^5^ Pa	5.72	5.63	4.91

**Table 2:** The changes in normalized frequency corresponding to third harmonics (24 MHz), Δ*f*, changes in mass density, Δ*m/A,* determined from Sauerbrey equation, thicknes, *h*, and surface density, *σ*, of the cyt c layer covalently attached to the MUA. The thickness has been calculated based on the density of the cyt c: 1.35 g/cm^3^ [44]. Results are mean ± SD obtained from three independent experiments.

Changes in Frequency and Surface Density	Cyt C, μM			
	0.5	2	4.5	10
Δ*f*, Hz	−21.16 ± 6.48	−33.60 ± 6.95	−23.30 ± 3.25	−22.66 ± 2.31
Δ*m/A*, ng/cm^2^	146.0 ± 44.7	231.8 ± 48.0	160.8 ± 22.4	156.35 ± 15.9
*h*, nm	1.08 ± 0.33	1.72 ± 0.36	1.19 ± 0.17	1.16 ± 0.12
*σ*, 10^12^ cm^−2^	7.33 ± 2.24	11.64 ± 2.41	8.10 ± 1.13	7.84 ± 0.80

## Data Availability

Not applicable.

## Supplementary Materials

The following supporting information can be downloaded at: https://www.mdpi.com/article/10.3390/bios13020251/s1, Figure S1: Electron microscopy image of AuNWs; Figure S2: Interaction of the gold nanowires with cyt c covalently immobilized at the surface of MUA chemisorbed at gold layer of the quartz crystal; Figure S3: Interaction of the DNA aptamers with cyt c covalently immobilized at the surface of MUA chemisorbed at gold layer of the quartz crystal.
