# Supplementary material for: Analysis of the Interaction between DNA Aptamers and Cytochrome C on the Surface of Lipid Films and on the MUA Monolayer: A QCM-D Study
Source: Biosensors (Basel). 2023 Feb 9;13(2):251. doi: 10.3390/bios13020251 (PMC9953847; doi:10.3390/bios13020251)
Supplement: Supplementary file 1 [file biosensors-13-00251-s001.zip › biosensors-2176126-supplementary.pdf]

biosensors

MDPI

Supporting Information

Analysis of the Interaction Between DNA Aptamers and Cytochrome C on the Surface of Lipid Films and on the MUA Monolayer: A QCM-D Study †

Marek Tatarko, Sandro Spagnolo, Martin Csiba, Veronika Šubjaková and Tibor Hianik *

Faculty of Mathematics, Physics and Informatics, Comenius University, Mlynská dolina F1, 842 48 Bratislava, Slovakia

* Correspondence: tibor.hianik@fmph.uniba.sk

† This paper is an extended version of our paper published in Tatarko, M.; Spagnolo, S.; Hianik, T. Monitoring of cytochrome c adsorption at supported lipid membranes using multiharmonic QCM method. In *Proceedings of the 2nd International Electronic Conference on Biosensors, 14–18 February 2022*; MDPI: Basel, Switzerland, doi:10.3390/IECB2022-12271.

S1. Electron microscopy image of AuNWs

Gold nanowires (AuNWs) were observed with standard Secondary Electron detector and in transmission mode with a STEM detector using Electron microscope EVO LS15 (Zeiss, Oberkochen, Germany) with following parameters: accelerating voltage 30 kV, Iprobe 100 pA. Length size of AuNWs was evaluated as 3.22 ±0.38 μm using ImageJ software.

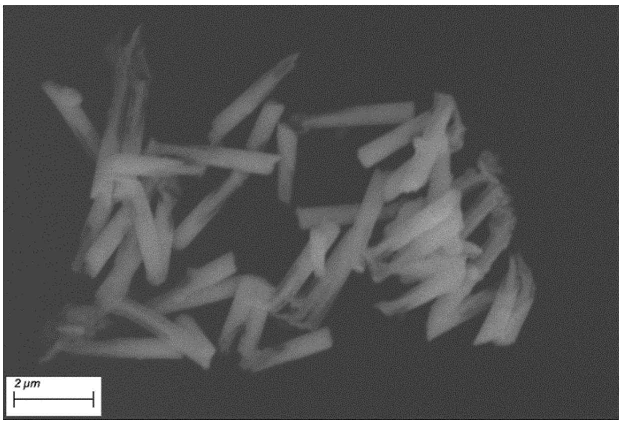

Figure S1. Electron microscope image observed with standard Secondary Electron detector of synthesized AuNWs.

S2. Interaction of the gold nanowires with cyt c covalently immobilized at the surface of MUA chemisorbed at gold layer of the quartz crystal

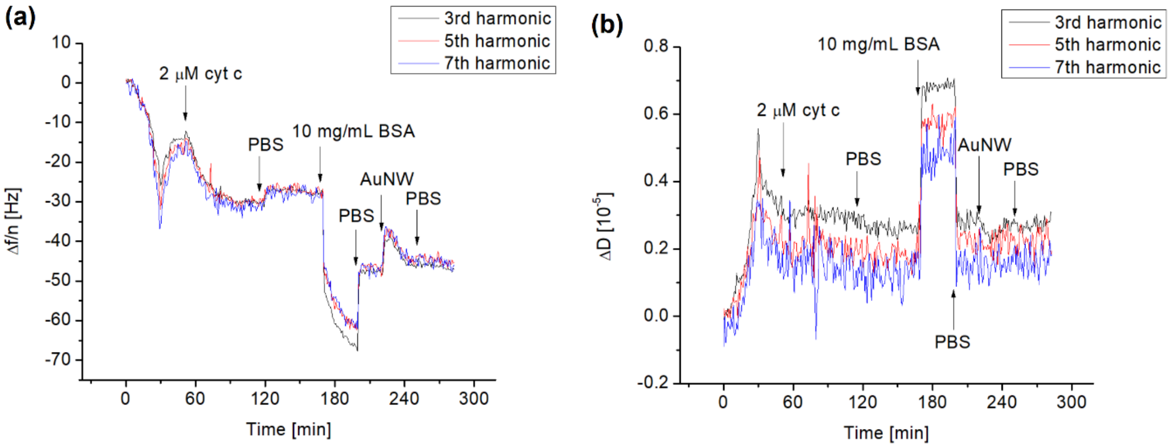

Figure S2. The kinetics of the changes of normalized resonant frequency (**a**) and dissipation (**b**) for 3rd, 5th and 7th harmonic frequencies vs. time following addition to the MUA surface of 2 μM cyt c, 10 mg/mL BSA and unmodified gold nanowires (AuNW). The moments of addition of cyt c, BSA, AuNW and PBS wash are shown by arrows.

S3. Interaction of the DNA aptamers with cyt c covalently immobilized at the surface of MUA chemisorbed at gold layer of the quartz crystal

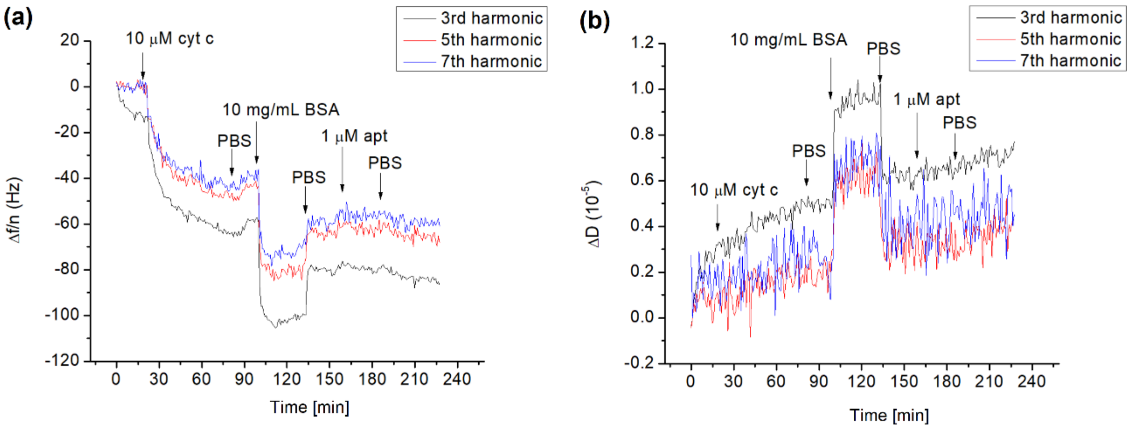

Figure S3. The kinetics of the changes of normalized resonant frequency (**a**) and dissipation (**b**) for 3rd, 5th and 7th harmonic frequencies vs. time following addition to the MUA surface of 2 μM cyt c, 10 mg/mL BSA and DNA aptamers specific to cyt c (apt). The moments of addition of cyt c, BSA, aptamer and PBS wash are shown by arrows.
